# Investigation of structural, electronic and magnetic properties of breathing metal–organic framework MIL-47(Mn): a first principles approach†

**DOI:** 10.1039/c9ra09196c

**Published:** 2020-01-29

**Authors:** Mohammadreza Hosseini, Danny E. P. Vanpoucke, Paolo Giannozzi, Masoud Berahman, Nasser Hadipour

**Affiliations:** Department of Physical Chemistry, Tarbiat Modares University Tehran Iran Hadipour@modares.ac.ir; UHasselt, Institute for Materials Research (IMO-IMOMEC) Agoralaan, 3590 Diepenbeek Belgium; IMOMEC, IMEC vzw 3590 Diepenbeek Belgium; Dipartimento di Scienze Matematiche, Informatiche e Fisiche, Università degli Studi di Udine Via delle Scienze 208 33100 Udine Italy; CNR-IOM DEMOCRITOS, SISSA Trieste Italy; Department of Electrical and Computer Engineering, Advanced Graduate University of Technology Kerman Iran

## Abstract

The structural, electronic and magnetic properties of the MIL-47(Mn) metal–organic framework are investigated using first principles calculations. We find that the large-pore structure is the ground state of this material. We show that upon transition from the large-pore to the narrow-pore structure, the magnetic ground-state configuration changes from antiferromagnetic to ferromagnetic, consistent with the computed values of the intra-chain coupling constant. Furthermore, the antiferromagnetic and ferromagnetic configuration phases have intrinsically different electronic behavior: the former is semiconducting, the latter is a metal or half-metal. The change of electronic properties during breathing posits MIL-47(Mn) as a good candidate for sensing and other applications. Our calculated electronic band structure for MIL-47(Mn) presents a combination of flat dispersionless and strongly dispersive regions in the valence and conduction bands, indicative of quasi-1D electronic behavior. The spin coupling constants are obtained by mapping the total energies onto a spin Hamiltonian. The inter-chain coupling is found to be at least one order of magnitude smaller than the intra-chain coupling for both large and narrow pores. Interestingly, the intra-chain coupling changes sign and becomes five times stronger going from the large pore to the narrow pore structure. As such MIL-47(Mn) could provide unique opportunities for tunable low-dimensional magnetism in transition metal oxide systems.

## Abstract 2

The structural, electronic and magnetic properties of the MIL-47(Mn) metal–organic framework are investigated using first principles calculations.

## Graphical abstract

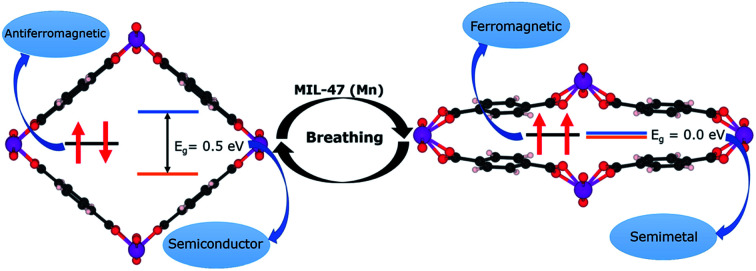

## Introduction

Metal–Organic Frameworks (MOFs) are a class of novel crystalline materials composed of metal(-oxide) nodes and organic linker molecules^1–3^ This combination of nodes and linkers results in a crystal lattice with amazing properties such as high internal surface area, porosity and chemical^4^ and physical tunability.^5^ Due to these unique properties, MOFs are being developed in applications such as gas storage,^6–8^ water filtering,^9,10^ catalysis,^11,12^ photocatalysis,^13^ drug delivery,^14,15^ energy storage^16^ and also their luminescence properties have been attractive for some applications.^17,18^ MOFs are also exceedingly interesting for fundamental research focusing on exotic and low dimensional physics in materials science.^19^ Breathing MOFs are a subclass of MOFs having the ability to reversibly change their unit cell volume by as much as 50% under changes in temperature,^20^ guest molecules,^21^ or external pressure.^22^ Also, this volume change is reported for MOFs out of the breathing class.^23^ Several comprehensive reviews on their properties and applications are available in the literature,^24,25^ reporting the existence of multiple breathing MOFs: MIL-53(Al),^26^ COK-69(Ti),^27^ SHF61.^28^ Of these, several appear to have a MIL-47/MIL-53 type topology with different transition metal nodes. Much theoretical and experimental work is being performed to study their intrinsic properties and possible potential applications.^29–31^ In particular, the spin configuration was linked to the sample type: single crystal samples present mainly antiferromagnetic chains, while powder samples show mainly ferromagnetic configurations.^32^ The very weak inter-chain spin-coupling makes the MIL-47(V) also an interesting quasi-1D material.^31^ The small energy differences and barriers between different structures make their study very challenging, even for modern high-accuracy DFT calculations.^33^ Similar as the UiO-66(Zr),^34^ the electronic structure of the MIL-47(V) can be tuned by means of linker functionalization.^4^ In their theoretical study of a series of breathing MIL-53(X), with X = Fe, V, Sc, Cr, In, Ga, and Al, Ling and Slater observe a significant change of electronic properties, in particular of the band gap, as a result of the large-pore to narrow-pore transition.^35^ The electronic and magnetic properties of DUT-8 (Ni), another flexible MOF with MIL-47 topology, were investigated by Trepte *et al.*^36^ Their results show that the magnetic ordering of nodes can be tuned by altering the ligands which coordinate to the transition metals.

Manganese is one of the elements with a very rich variety of electronic and magnetic properties in solid-state materials.^37,38^ Munn *et al.* investigated Mn-based MOFs with a MIL-47 topology, containing both 1,4-benzenedicarboxylate (BDC) and pyridine-*N*-oxide (PNO) linkers. Although the MOF is paramagnetic at room temperature, they observe anti-ferromagnetic behavior at low temperature.^39^ A high capacity electrode Mn-based material with high performance in energy storage field was introduced by Liu *et al.*^40^ and an ultra-layered Mn MOF was found to be a good candidate for lithium storage.^41^ The remarkable properties of breathing MOFs combined with the versatility of Mn, which is shown theoretically in previous literature,^42^ motivate us to study a breathing Mn-based MOF: MIL-47(Mn). Recently a new MOF, Mn(ii) unsaturated metal nodes and BDC linker, has been synthesized and its structural change toward water adsorption was observed.^43^ It may be promising for future synthesis of MIL-47(Mn).

In this work, the structural, electronic and magnetic properties of the compound, MIL-47(Mn) are calculated for both of large (LP) and narrow pore (NP) morphology using periodic density functional theory (DFT). We compare the results for MIL-47(Mn) with MIL-47(V) examining in detail differences and similarities.

## Computational details

### DFT calculations

DFT calculations,^44^ in particular periodic DFT calculations with a plane wave basis set, are increasingly used to study MOFs.^45,46^ The advantage of a plane wave basis set is its ability to optimize the atomic positions as well as the shape and volume of the unit cell, while being unbiased and free from basis set superposition error.^47^ All calculations have been performed within DFT as implemented in the Quantum ESPRESSO (QE) package.^48^ We use ultrasoft pseudo-potentials, and the Perdew, Burke and Ernzerhof (PBE)^49^ exchange–correlation functional. Previous studies have shown that dispersive interactions (*c.q.*, van der Waals forces) are crucial for the accurate optimization of the atomic structure and the relative stability of MOFs, especially in breathing ones.^50^ The DFT-D2 correction, formulated by Grimme *et al.*^51^ and implemented in the QE package, was applied to obtain more reliable structures. The kinetic cut-off energy for plane waves is set to 475 eV. The first Brillouin zone is sampled using a 2 × 2 × 6 and 2 × 6 × 6 Monkhorst–Pack *k*-point grid for LP and NP, respectively. The total and partial density of states are computed using a denser sampling grid of 3 × 3 × 9 and 3 × 9 × 9 *k*-points for LP and NP, respectively. Atomic charges are calculated from the electron density, using the Hirshfeld-I atoms-in-molecules partitioning scheme, which is know to provide consistent and high quality atomic charges in porous materials^52–54^ A spherical Lebedev grid of 1202 grid points is used in combination with a logarithmic radial grid.^55,56^ The threshold for convergence was set to 1.0 × 10^−5^ electron.

### Structural model of the MIL-47(Mn)

**Fig. 1:**
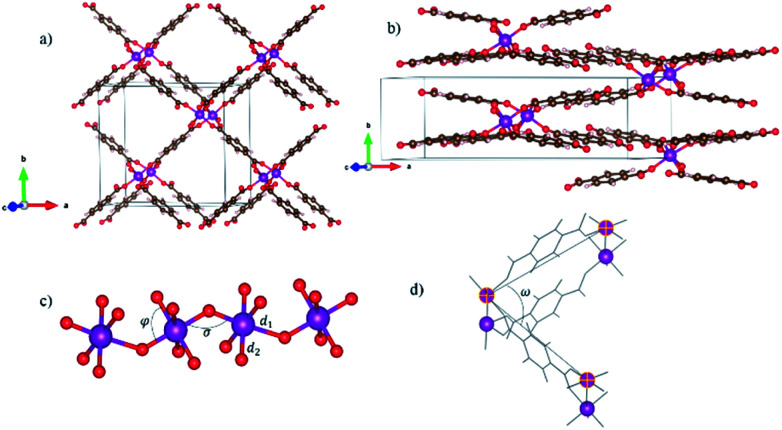
Structure of MIL-47(Mn): (a) large pore (b) narrow pore (c) metal oxide chain in which all of the inequivalent Mn–O bonds and octahedral related parameters are shown (d) pore angle. Atomic species: violet Mn, red O, brown C, white H atoms.

The initial atomic structures were constructed starting from the crystallography files for both the LP and NP MIL-47(V) CCDC 1419980 and 1419981,^33^ respectively. In these structures the V atoms were replaced by Mn. All schematic presentations of materials are done using the VESTA^57^ visualization software. In order to study the magnetic properties, spin-polarized calculations were performed. The Mn atoms were considered as the magnetic centers and different configurations were studied for both LP and NP. Fig. 1 shows typical ball-and-stick representation of our system.

Due to the relatively flat potential energy surface (PES) of breathing MOFs, accurate structure optimization can be a challenging task.^33^ We have therefore taken a two-step approach. In our first step, the geometry is optimized without taking the spin configuration into account, as it was shown, in case of the MIL-47(V), that the local spin configuration has only a minor influence on the geometry of the system.^31^ For the LP, first the atomic positions of MIL-47(Mn) were optimized at fixed volume. Then, a variable-cell relaxation was performed, in which both the atomic positions and the cell vectors were optimized. Also, the same procedure was performed for NP version. The resulting LP configuration had an orthorhombic lattice while the NP configuration had a monoclinic one.

**Fig. 2:**
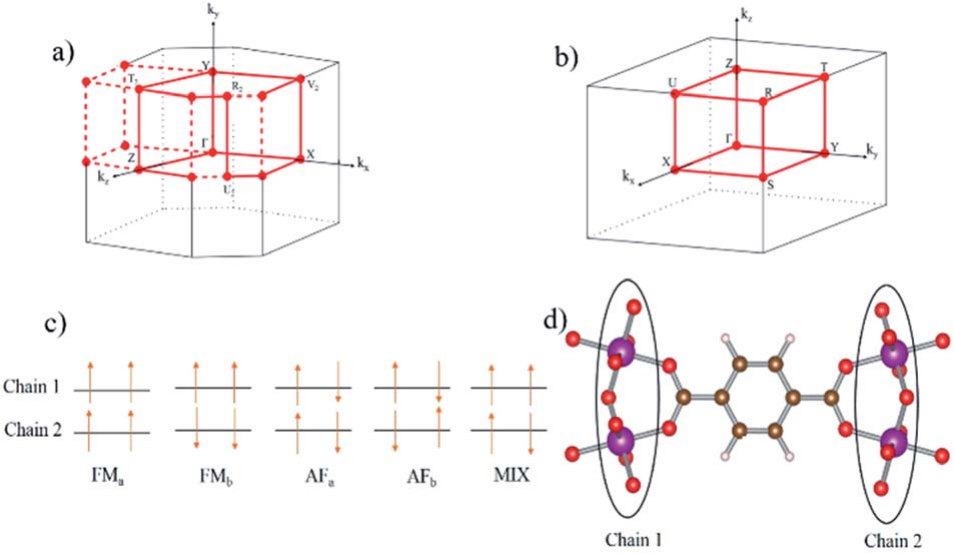
Reciprocal lattice and different high symmetry points of (a) monoclinic and (b) orthorhombic lattice is used to calculate the band structure for NP and LP geometry respectively. (c) Different magnetic configurations of our structure (d) magnetic centers in real space of two different Mn–O chains.

Starting from these pre-converged geometries, spin-polarized structure optimizations were performed, as a second step, to obtain the geometries of the five possible inequivalent spin configurations. These spin configurations are schematically represented in Fig. 2: FM_a_ and FM_b_ (ferromagnetic chains), AF_a_ and AF_b_ (antiferromagnetic chains), and MIX (50% ferromagnetic and 50% antiferromagnetic chains). For each spin configuration, both fixed and variable cell optimizations are performed and compared. Due to nearly flat PES, the variable-cell relaxations are not sufficiently accurate for breathing MOFs, in agreement with earlier findings.^33^

The energetically most stable structure obtained in the fixed volume calculations, AF_a_, was selected and the optimal volume for the AF_a_ spin configuration was obtained through a fitting to the Murnaghan equations of state (EOS).^60^ Then, starting from this structure, the atomic positions for the different spin configurations were obtained by optimizing the geometry under fixed volume constraints, after which the electronic and magnetic properties of the different spin configurations were computed. The difference between volumes obtained through EOS-fitting and variable-cell optimization is very small (∼0.05%). Therefore, we used the computationally less expensive variable-cell optimization for the NP.

## Results and discussions

### Structural properties

The cell parameters of the LP and NP structures are listed in Table 1. The computed volume is smaller than for MIL-47(V), which is used as initial geometry. This difference in volume originates mainly from the shorter *c*-axis. Table 1 indicates some structural features for MIL-47(Mn) like materials, MIL-53(Al)^58^ and (Ga)^59^ which have been reported experimentally. One can find MIL-47(Mn) has been optimized in smaller volume than them for both LP and NP crystal phase. As shown in Table 1, the breathing behavior results in a drastic change in volume. In addition, the symmetry changes from orthorhombic (LP) to monoclinic (NP). Such a volume change has been found to significantly alter physical and chemical properties such as the electronic structure.^35^ Let us therefore focus on the MnO_6_ complex and the metal-oxide chain part of the unit cell. Table 2 shows the energy and structure parameters of different magnetic configurations for both LP and NP phases. The global and local geometry of the MIL-47(Mn) MOF is shown in Fig. 1.

**Table 1:** Cell parameters of the different crystal phases studied and available experimental data for MIL-47 like structures. LP: large pore, NP: narrow pore. Lattice parameters are in Å, volume is in Å^3^. For MIL-47(V) data is taken from the CIF files

Structure	*a*	*b*	*c*	*α*	*β*	*γ*	Volume
LP-MIL-47(Mn)	16.20	13.88	6.50	90	90	90	1460.70
NP-MIL-47(Mn)	20.98	6.51	6.44	90	113	90	806.76
LP-MIL-47(V)^33^	16.23	13.97	6.85	90	90	90	1553.12
NP-MIL-47(V)^33^	21.11	6.84	6.77	90	112.38	90	904.86
Exp-LP-MIL-53(Al)^58^	16.91	12.67	6.62	90	90	90	1419.0
Exp-NP-MIL-53(Al)^58^	20.82	6.87	6.61	90	119.95	90	863.9
Exp-LP-MIL-53(Ga)^59^	16.68	13.21	6.72	90	90	90	1479.7
Exp-NP-MIL-53(Ga)^59^	19.83	6.86	6.71	90	103.88	90	886.3

Comparing the relative energies for each of the magnetic configurations, we note that the spin ground state changes from an anti-ferromagnetic configuration to a ferromagnetic one upon collapse of the pores (LP to NP transition). The anti-ferromagnetic ground state for the LP phase makes this system similar to the MIL-47(V), while the FM ground state for the NP makes it different from the MIL-47(V). The relative energies, listed in Table S1,† indicate that the energy of transition between different magnetic configurations for LP is much smaller than for the phase transition between LP and NP. Here, we focus on the differences in geometry of distinct magnetic structures. We found that the magnetic configuration can affect some structural parameters, especially metal-oxide clusters ones.

Generally, all clusters show deviations from a perfectly octahedral complex, due to the presence of different ligands around the Mn atom (O^2−^ in axial direction and BDC^−^ in plane of complex). Mn–O bonds can be divided into two categories: the bonds d_2_ between Mn and the oxygen atoms of the BDC organic linkers, forming the plane of the octahedral complex, and the bonds d_1_ between Mn and oxygen atoms of metal oxide chains (*cf.*Fig. 1). Interestingly, in contrast to MIL-47(V)—where two different bond lengths along *c*-axis had been reported,^31^ we observe only one bond length along the MnO chain. As a result, we also find a volume difference between MIL-47(Mn) and MIL-47(V).

The +4 oxidation state of the metal in the MIL-47 framework leaves the V and Mn with one and three unpaired electrons in t_2g_ orbitals, respectively. So, the presence of a Jahn–Teller effect is expected for the V version of MIL-47 in addition to a larger volume compared to MIL-47(Mn). The obtained length for d_1_ is in good agreement with previous works, presenting a length typically observed in bulk manganese oxide phases.^61^ On the other hand, d_2_ length is sufficiently close to the obtained value for the Mn–O bond length in organometallic environments.^62^

As shown in Table S1,† there is a difference of almost 0.2 angstrom between them. This difference in bond length results from the different strength of two ligands O^2−^ and BDC^−^ which induce different fields of strength. To characterize the MnO chains further, we study two relevant angles of the MnO_6_ complexes (*cf.*Fig. 1). First, the “super-exchange” angle *σ*, is defined as the Mn–O–Mn angle in the metal-oxide chain; the second, the O–Mn–O angle, *φ*, is part of octahedral complex. The super-exchange angle obtained for NP is smaller than for LP. Also, in contrast to MIL-47(V) the ferromagnetic configuration results in smaller *σ* in both crystal structures which can be originated from difference in number of unpaired electrons between Mn and V. This phenomena could be investigated in terms of the Goodenough–Kanamori rules.^63^ In this context, super-exchange angles *σ* = 90 and 180 degrees are indicative of ferromagnetic and antiferromagnetic coupling, respectively.

Accordingly, in this research antiferromagnetic coupling was observed for the larger angles, while the smaller ones show the ferromagnetic behavior. The angle *φ* of the octahedral complex is nearly identical for both LP and NP, and present a small deviation from the perfect octahedral configuration in which this angle is 90°.

As mentioned before, breathing occurs through the change of cell shape and volume. The opening angle, *ω*, is defined as the angle made by three Mn atoms and follows the pore opening (*cf.*Fig. 1d) angle can be considered as a quantitative parameter for breathing MOFs and has been computed as 81.2° and 37.2° for LP and NP, respectively which is within 1° of the opening angles for LP and NP configurations of the MIL-47(V).^33^ It should be noted that although DFT+U could be considered as a suitable functional correction for describing the electronic structure of transition metal oxides, the U-dependence of other materials properties, including the lattice parameters, makes it less suited for the investigation of the atomic structure. With regard to the system at hand, previous reports on manganese-oxide compounds have shown that PBE+U tends to overestimate some structural features.^64^

### Electronic properties

**Fig. 3:**
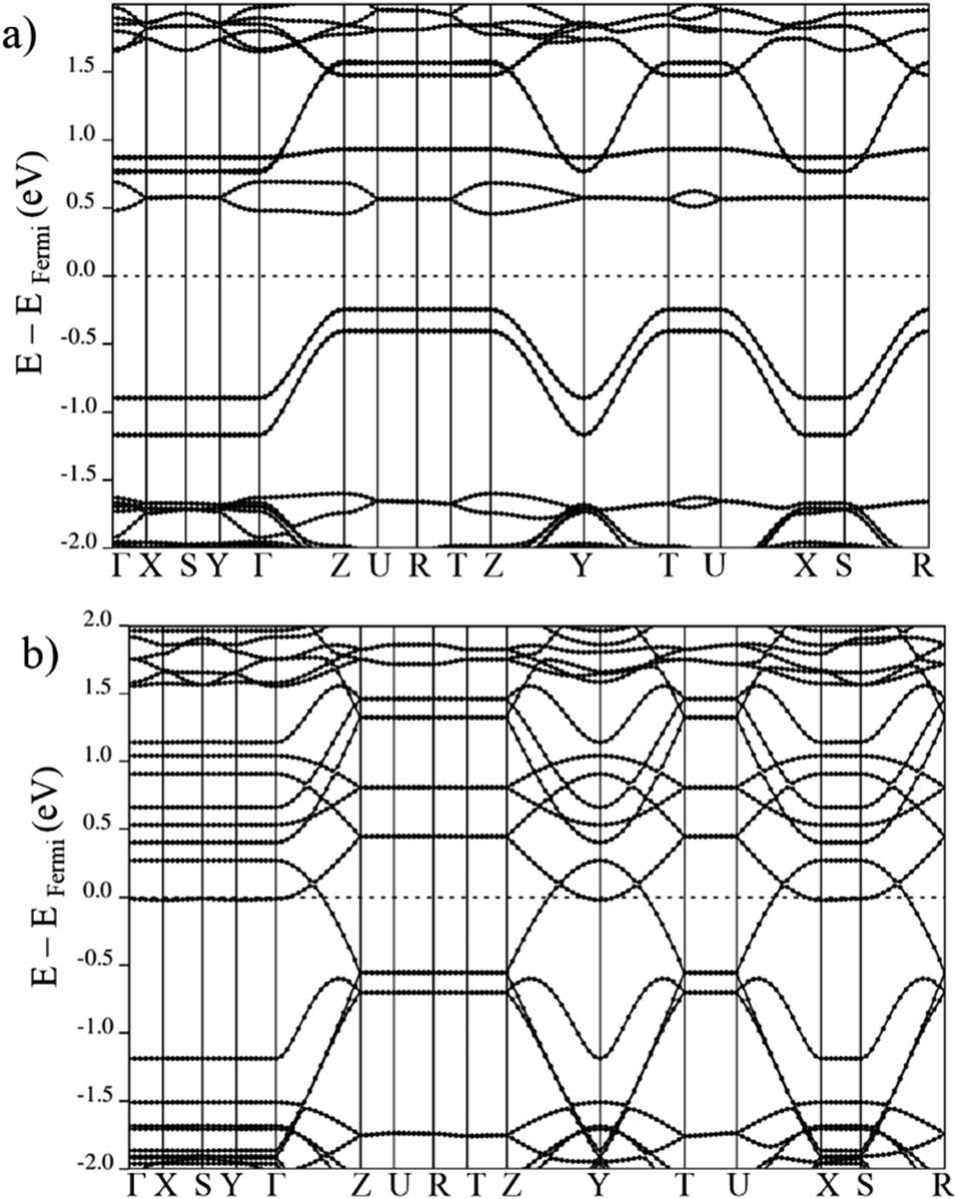
Electronic band structure of large pore geometry in (a) AF_a_ (b) FM_b_ magnetic configuration around Fermi level.

The electronic band structures for all magnetic configurations are calculated along the high-symmetry lines of the first Brillouin zone of LP and NP structures, shown in Fig. 2. Densities of states (DOS) and DOS projected on atomic orbitals (PDOS) are reported for both of large and narrow pore which crystallize into orthorhombic and monoclinic lattice systems, respectively. With our unit cell containing four Mn centers, five inequivalent magnetic configurations are possible (*cf.*Fig. 2). Although the ground state is antiferromagnetic AF_a_, investigation of other spin configurations may be worthwhile to gain a deeper understanding of the electronic behavior. In the case of MIL-47 topology, the coordination environment requires a +4 oxidation state of the transition metal. The electronic structure of Mn^4+^ contains three unpaired d electrons, giving rise to a 3/2 spin per Mn center. The PDOS of 4s and 3d with different m_l_ numbers for Mn of LPAF_a_ (presented in S5†) confirm the Mn^4+^ configuration. Fig. 3 presents the electronic band structure of the zero total-magnetization configurations, FM_b_ and AF_a_ systems, in the LP geometry (AF_b_ is similar to AF_a_ so this is presented in ESI†). One immediately notices an important difference between these two systems. The AF_a_ configuration presents a small bandgap, while in the FM_b_ system several bands cross the Fermi level, making it a metallic system. A similar behavior is observed for AF_b_, shown in Fig. S1,† and FM_a_, presented in Fig. 5. For the ferromagnetic configurations, the valence bands cross the Fermi energy along Γ–Z, Z–Y, Y–T and U–X, and S–R lines which all are oriented along the *c*-axis of the system (Mn–O chains). Along the orthogonal directions the bands near the Fermi level are almost perfectly flat, indicative of quasi-1D behavior, similar to what was observed for the MIL-47(V) system.^31^ There are minor differences between FM_a_ and FM_b_ spin up configurations (but also between AF_a_ and AF_b_), showing that some coupling between the chains is present but it is small. Both antiferromagnetic systems have the same band gap, about 0.5 eV, at the same position in the first Brillouin zone. Closer comparison with the electronic band structure of MIL-47(V) shows, in case of the antiferromagnetic configurations, the presence of two almost parallel bands just below the Fermi-level. These bands are associated with the additional unpaired d electrons of Mn^4+^ (when compared to V^4+^).

**Fig. 4:**
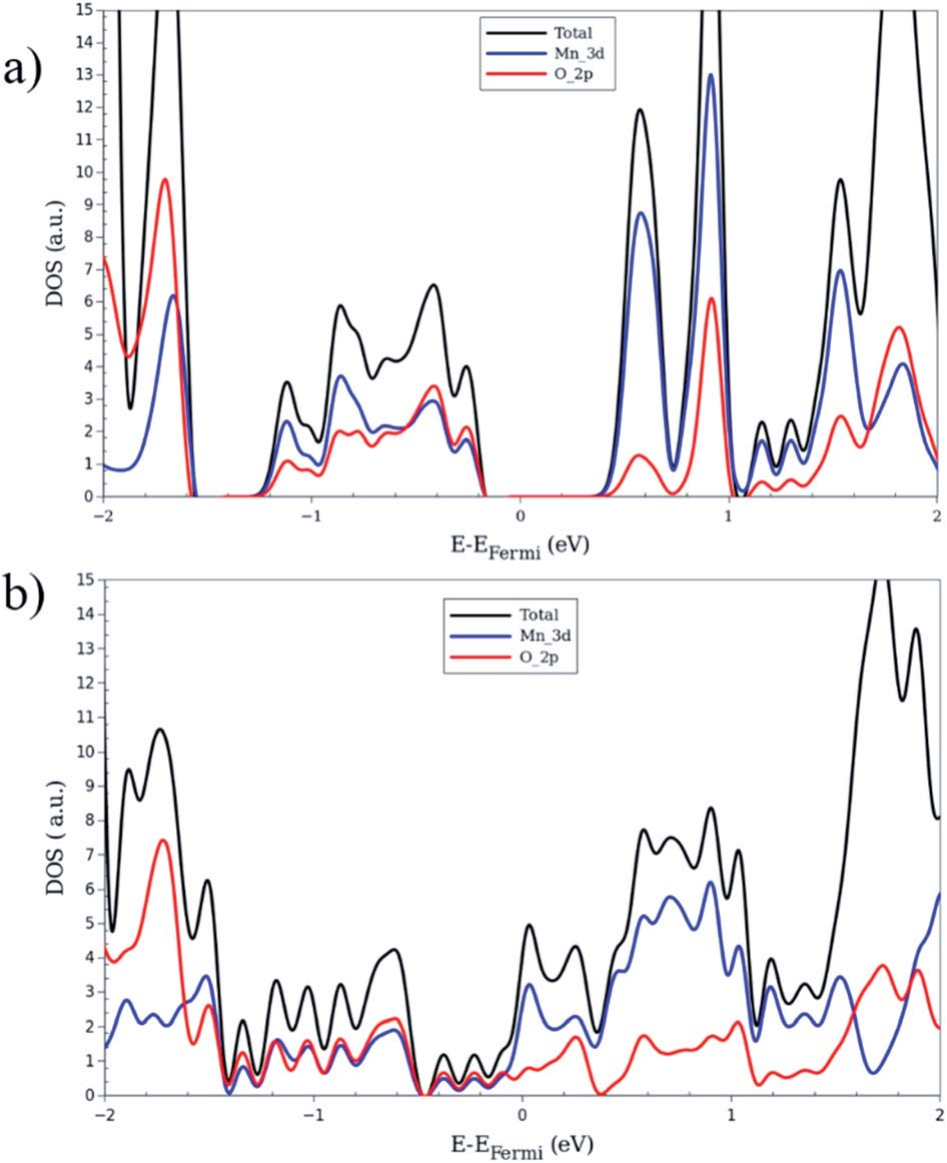
Total and projected density of states of large-pore geometry in (a) AF_a_ (b) FM_b_ magnetic configuration. The black, blue and red lines represent total, Mn 3d and O 2p contributions respectively.

**Fig. 5:**
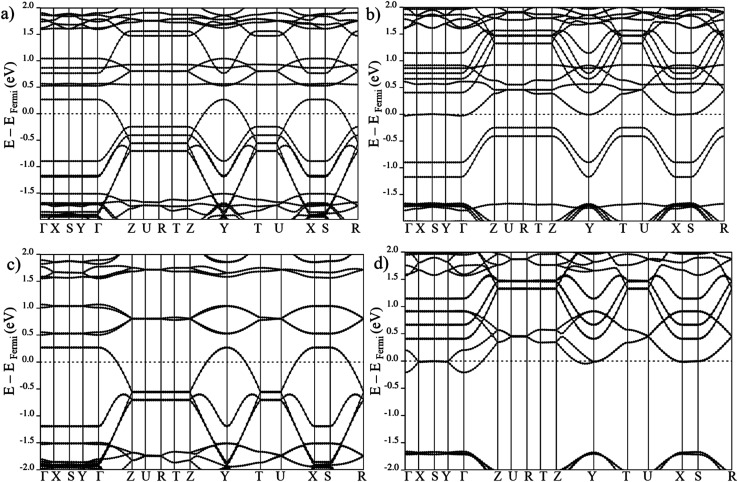
Electronic band structure of large pore geometry in (a) MIX spin up, (b) MIX spin down, (c) FM_a_ spin up and (d) FM_a_ spin down magnetic configuration around Fermi level.

On the other hand, in case of the ferromagnetic spin configuration, with non-zero total magnetization (MIX and FM_a_), a remarkable difference between majority and minority spin is observed. In the case of MIX (*cf.*Fig. 5), the valence band in the spin up crosses the Fermi level but for spin down the conduction band with the Fermi level. Same behavior is seen for the FM_a_ configuration with the down spin conduction band coinciding with the Fermi level while the spin up valence band crosses it. In the FM_a_ case, the degeneracy of the coinciding conduction band is even lifted by weak in-plane coupling of the Mn centers of neighboring chains.

To investigate the electronic structure from a different perspective, the DOS and PDOS are calculated for all configurations. Fig. 4 shows them for FM_b_ and AF_a_ as representative systems of zero total magnetization, Fig. S3† exhibits the plots for MIX and FM_a_ of non-zero magnetization case. For the systems with zero global magnetization: FM_b_, AF_a_ and AF_b_, the spin-up and spin-down DOS's are identical so only the up component is shown. Comparison of Fig. 4 and S2† shows that AF_a_ and AF_b_ present a nearly identical DOS, in stark contrast with the FM_a_ and FM_b_ systems, which results from the difference in the global magnetization of the latter. A near identical DOS can be an additional indication for weak inter-chain interaction. In order to obtain a more detailed insight in the electronic properties of the LP structures, we consider the projection of the DOS onto atomic orbitals. The main contribution around the Fermi level comes from the metal-oxide complex. For this reason, the PDOS analysis is only performed for Mn and O atoms. In Fig. 4, it can be seen that the valence band contains nearly the same contribution for both Mn_d_ and O_p_ electrons, showing a strong hybridization between them and formation of chemical bonding. In contrast, the conduction band presents mainly a Mn_d_ character. Looking at the band diagram for AF_a_ and MIX into detail, some nearly flat bands are observed along the whole Brillouin zone, in the energy range from 0.5 to 1.0 eV above the Fermi level. These give rise to the sharp Mn_d_ peak in the corresponding DOS plots.

**Fig. 6:**
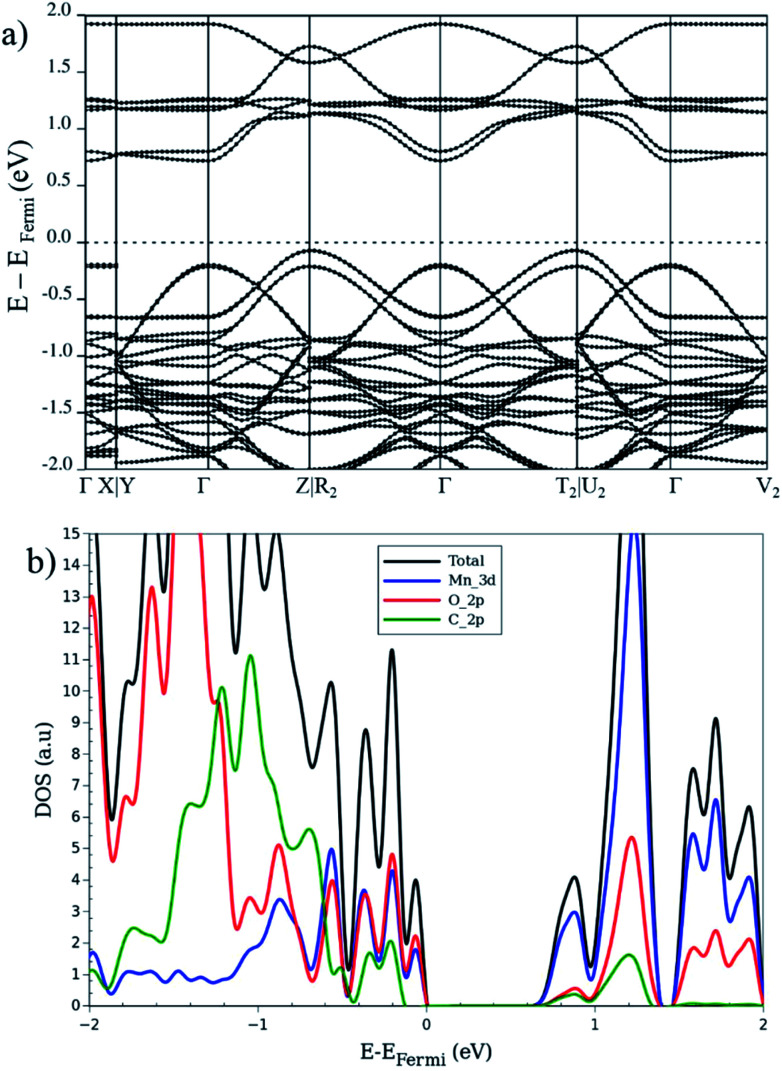
(a) Band structure, (b) total and projected DOS, for the narrow-pore geometry in the AF_b_ magnetic configuration.

So far, the electronic properties of the LP in different magnetic configurations were described. Let us now focus on the electronic features of the NP structure. Ling and Slater^35^ noted that the band gap of a NP is usually reduced with respect to the LP, in case of MIL-53. This reduction is attributed to a smaller volume and a larger interaction between organic linkers. In this context, we investigate the AF_b_ and FM_a_ magnetic configurations for the NP as two case of studies. Fig. 6 depicts the band structure, DOS and the projected (PDOS) for AF_b_ magnetic configuration of NP crystal. In contrast to the LP, the NP DOS also presents a significant C_p_ character in addition to the O_p_ and Mn_d_ contributions near the Fermi level especially in valence band region. As a result of the crystal volume reduction and of the increase of interactions between the organic linkers, the contribution of carbon atoms around Fermi level is enhanced compared to the LP.

From band structure analysis, in contrast to the same configuration in LP, no flat bands in valence region can be seen and electronic dispersion is present throughout the first Brillouin zone. On the other hand, for the FM_a_ configuration, like in the same configuration for LP, the band diagram for the spin-up states crosses the Fermi level and a band gap, about 1.2 eV at the Γ point appears for the spin-down states. In the latter structure, the carbon contribution is not observable below the Fermi level and only appears above it. In contrast, the AF_a_ and AF_b_ configurations present a larger contribution of carbon atoms both below and above the Fermi level. In case of the FM_a_ and MIX configurations with a global non-zero magnetization, the up and down spins show remarkable differences. The electronic band structure of AF_b_ presents a strong dispersion of the valence electrons along the high-symmetry lines of the first Brillouin zone for the NP (*cf.*Fig. 6). In contrast, for FM_a_, as shown in Fig S4,† a flat band in the *XY* plane is seen while the dispersion along the paths parallel to *c*-axis remain. Furthermore, the conduction band is a combination of several (almost entirely) flat bands originated from Mn_d_, O_p_ and C_p_ electrons. This dependency of electronic properties on the magnetic configuration makes the investigation of the spin–orbit coupling within this MOF of interest for future study. Recently, spin spirals have been observed in manganese oxide chains experimentally^65^ and confirmed by theoretical methods. This behavior can also occur for the MIL-47(Mn) system due to presence of 1-D Mn–O chain. However, this is beyond the scope of the current study and will be considered for future work.

### Charge density analysis

**Fig. 7:**
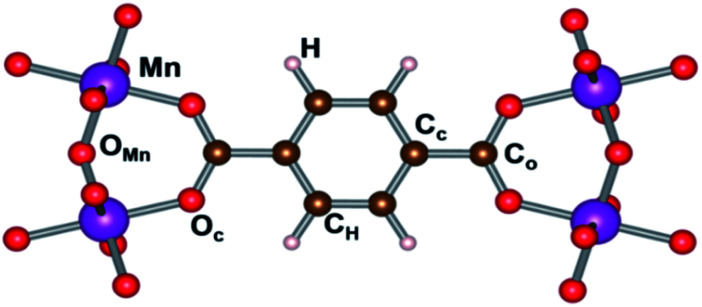
Inequivalent atomic positions in MIL-47(Mn).

Hirshfeld-I charges were calculated for the different spin configurations of the LP and NP geometry of the MIL-47(Mn).^52,53^ The different inequivalent sites are indicated in Fig. 7, while average values are presented in Table S2.† The standard deviation on these values is, with the exception of the H atoms in the NP geometry, below 0.01*e*, and in most cases even below 0.001*e*. As can be seen in Table S2,† the average charges show no significant variation between the various spin configurations, indicating the different spin configurations do not give rise to a different local chemistry. Even more, also the transition of the NP to the LP has only minor impact on the atomic charges. The largest relative variation is seen in the atomic charge of the C_c_ atoms (about 7%), which could be related to the bending of the terephthalate linker.

Interestingly, if we compare the charge of the Mn to that of V in a MIL-47(X) topology, we see that both elements have nearly the same charge, with the Mn one, slightly smaller than the 2.44*e* observed for V, supporting the assumption Mn to be in a +4 oxidation state.^31^ In contrast, the O ions of the metal-oxide chain have a significantly larger negative charge of −1.16*e* of O_Mn_ instead of −1.01 for O_V_ in MIL-47(V), while the O atoms in the plane of the MnO_6_ octahedra have the same charge as in the VO_6_ case. This shows the O_c_ and O_Mn_ atoms present a different bonding with the Mn atom. Furthermore, it shows the transition-metal oxide chains to have a stronger polarization in case of Mn, in line with the electronegativity of the elements. In contrast, the polarization between the linker and the transition metal oxide chain is significantly smaller in than for the MIL-47(V), with the linkers having a charge of about −1.25*e*, for MIL-47(Mn), compared to −1.41*e*, for MIL47(V).^33^ This shows us that the polarization within the framework can be tuned by selecting different metals. Furthermore, as charges in these MOFs are rather local, polarization gradients could be designed in mixed metal compounds.

### Magnetic properties

The magnetic properties of transition metals, owing to their unpaired electrons, are of great interest to chemistry and physics.^66,67^ In this context, MOFs can be viewed as a large lattice of organic linkers and widely separated transition metals centers. Such a configuration is very well suited for the observation of low-dimensional behavior. As the spin ground state is different for the LP and NP structures, this could also give rise interesting magnetic transitions. The computed local charges on the Mn centers of the different magnetic configurations (both LP and NP) indicate the same oxidation state is present for all configurations: in this case +4 which is typical one for MIL-47.

In our system, we define two coupling constants: the intra-chain coupling *J*_c_ and inter-chain coupling *J*_i_. We calculated the coupling constants using an Ising type model, eqn (1). Such a model was used successfully before with the MIL-47(V).^31^1
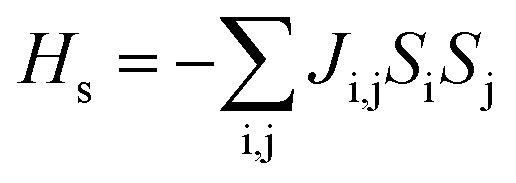
*J*_i,j_ represents the coupling constants between the i and j Mn-sites. *S*_i_ presents the site projected spin of site i. In this work, we use the theoretical value of 3/2 for all *S*_i_. We mapped the total energies of the 5 different spin-configurations on the model-Hamiltonian given in eqn (1), giving rise to a set of 5 equations. The set of 5 equations was numerically solved to obtain the two coupling constants.

The resulting coupling constants are listed in Table 2, for both LP and NP geometry.

**Table 2:** List of computed coupling constants obtained *via* DFT energy mapping onto Ising model

Structure	*J*_c_ (meV)	*J*_i_ (meV)
LP	−0.5808	0.0432
NP	2.8533	0.0749

There is a significant difference between the two crystalline structures from the point of view of magnetism. In comparison to MIL-47(V), we notice a significantly weaker interaction between magnetic centers.^31^ This indicates that MIL-47(Mn) can switch much easier than MIL-47(V) version between different magnetic configurations. The negative value of *J*_c_ of the LP structure is indicative of an antiferromagnetic ground state, corroborating our earlier observations. On the other hand, the NP shows a positive *J*_c_ value, indicating a ferromagnetic ground state. Quantitively, the computed intra-chain coupling value for the NP is about 6 times the one for LP, showing the intrachain spin coupling to strongly depend on the pore size despite the fact that the pore geometry has only a minor influence on the MnO-chain geometry. The inter-chain coupling is comparable for the NP and LP case, and at least one order of magnitude smaller than the intra-chain coupling, in agreement with quasi-1D magnetic behavior.

## Conclusion

In this work, structural, electronic and magnetic properties of MIL-47(Mn) as a breathing metal–organic framework was studied using periodic DFT calculations and the results were compared to its analogues, MIL-47(V). From the structural point of view, the obtained volumes for both large and narrow pore geometries were smaller than those reported for MIL-47(V), which can originate from the absence of Jahn–Teller effect in Mn. Also, MnO_6_ complex analysis shows that a deviation from octahedral symmetry, due to the presence of ligands with different field strength around the metal, can be found. Total energy data reveal an antiferromagnetic ground state for the large-pore geometry, but the system tends to move toward ferromagnetic state after breathing: the narrow-pore structure was found to have a ferromagnetic configuration. Electronic band structures show a strong dependency of electronic properties on spin configuration. In all cases, for both large- and narrow-pore crystals, the ferromagnetic configurations results in metallic behavior while the antiferromagnetic ones show semiconducting one. Also, a distinct behavior for bands parallel and orthogonal to the MnO chain is obtained. In the large-pore case, both valence and conduction bands show dispersion along the MnO chain and flat shape orthogonal to this. On the other hand, in narrow-pore geometry a dispersion can be detected even orthogonal to the MnO chain in the antiferromagnetic configuration. Calculated magnetic coupling constants confirm the stability of large- and narrow-pore structures. According to computed coupling constants, inter-chain interaction increases after volume reduction, but similarly to what reported in MIL-47(V)^31^ the inter-chain coupling is much smaller than intra-chain one, a reliable evidence for 1-D behavior in this material.

## Conflicts of interest

There are no conflicts to declare.

## Supplementary Material

RA-010-C9RA09196C-s001
