# Supplementary material for: Investigation of structural, electronic and magnetic properties of breathing metal–organic framework MIL-47(Mn): a first principles approach
Source: RSC Adv. 2020 Jan 29;10(8):4786–94. doi: 10.1039/c9ra09196c (PMC9049066; doi:10.1039/c9ra09196c)
Supplement: RA-010-C9RA09196C-s001 [file RA-010-C9RA09196C-s001.pdf]

Electronic Supplementary Material (ESI) for RSC Advances.
This journal is © The Royal Society of Chemistry 2020

Supporting in formation for "Investigation of structural, electronic and magnetic properties of breathing Metal-Organic Framework MIL-47 (Mn): a first principles approach "

Mohammadreza Hosseini[a], Danny E.P. Vanpoucke[b,c], Paolo Giannozzi[d,e], Masoud Berahman[f], Nasser Hadipour[a*]

[a] Department of Physical Chemistry, Tarbiat Modares University, Tehran, Iran

[b] UHasselt, Institute for Materials Research (IMO-IMOMEC), Agoralaan, 3590 Diepenbeek, Belgium

[c] IMOMEC, IMEC vzw, 3590 Diepenbeek, Belgium

[d] Dipartimento di Scienze Matematiche, Informatiche e Fisiche, Università degli Studi di Udine, via delle Scienze 208, 33100—Udine, Italy

[e] CNR-IOM DEMOCRITOS, SISSA, Trieste, Italy

[f] Department of Electrical and Computer Engineering, Advanced Graduate University of Technology, Kerman,Iran

S1. COMPUTATIONAL DETAILS

All calculations presented in this work are carried out using Quantum ESPRESSO simulation package. We use density functional theory with a plane-wave basis set with a kinetic energy cutoff of 475 eV. An ultra-soft pseudopotential was used for all atoms in the structure. GGA type exchange correlation functional formulated by Perdew-Burke-Ernzerhof , known as PBE was used to approximate exchange correlation effects. Since dispersion effects have a crucial contribution in MOF stability, the DFT-D2 correction was included in all calculations. The electronic temperature was set through the smearing method with gaussian smearing and 0.05 Ry width. First Brillouin zone sampling based on Monkhorst-Pack method was performed by 2x2x6 and 2x6x6 k-point grid. These grid formats were selected according to the cell vectors in both LP and NP geometry. The structural optimization was performed in the following way:

First, for LP, in the CIF files from MIL-47 (V) the Vanadium atoms were replaced by Manganese ones. An initial variable cell optimization, non-magnetic in this case, then a variable cell optimization on the output of previous step was done while the magnetic configurations were introduced. According to the previous studies, due to the presence of Pulay stress in MIL-47 (V) and to the nearly flat potential energy surface (PES), fitting on an equation of states (EOS) would be more reliable than variable-cell optimization to obtain optimized geometry. So, we took the most stable geometry, AFb, and performed the process of EOS fitting. Finally, at the obtained volume fixed-volume relaxations, in which only the atomic positions were allowed to be optimized, were performed for each magnetic configuration. We computed relative volume difference between output of variable cell and EOS fitting using equation s1, obtaining %DeltaV=0.05%.

$$\%\text{DeltaV} = \frac{V_{EOS} - V_{VC}}{V_{EOS}} \times 100 \qquad \text{(s1)}$$

Since we observed a tiny difference between volume obtained from EOS and variable cell relaxation, for NP we performed only variable cell relaxation and then at this fixed volume, atomic position optimization was done for each of magnetic configurations.

S2. Structural properties

Table S1. Detailed geometry data and corresponding energy for magnetic configurations of LP and NP structures. Bond lengths are in Å, energy differences in (meV/unit cell) and angles in degree

Structure	d_1	d_2	σ	φ	Relative Energy
LP FM_a	1.79	1.95	130.3	87.8	9.5
LP FM_b	1.79	1.95	130.3	87.1	11.3
LP AF_a	1.77	1.95	132.9	87.9	0.0
LP AF_b	1.77	1.95	132.9	87.8	0.0
LP MIX	1.79	1.95	132.9	87.8	4.1
LP non-magnetic	1.74	1.94	138	90.3	-
NP FM_a	1.79	1.96	127.6	87.4	108.8
NP FM_b	1.79	1.96	128.2	88.7	111.5
NP AF_a	1.77	1.96	130.3	88.3	160.5
NP AF_b	1.78	1.96	129.9	88.2	163.2
NP MIX	1.77	1.96	130.07	88.25	106.1
NP non-magnetic	1.74	1.95	134.5	89.6	-

S3. Electronic properties

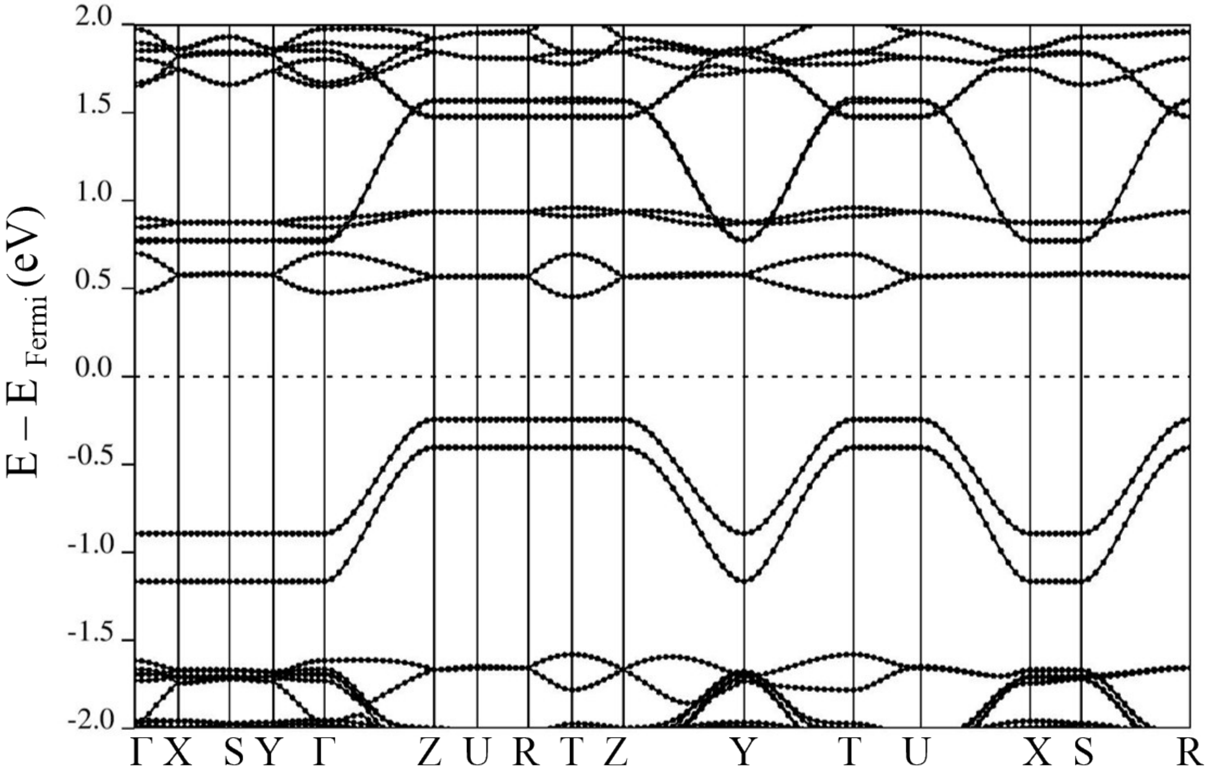

Figure S1. Electronic band structure of large pore geometry AF_b magnetic configuration

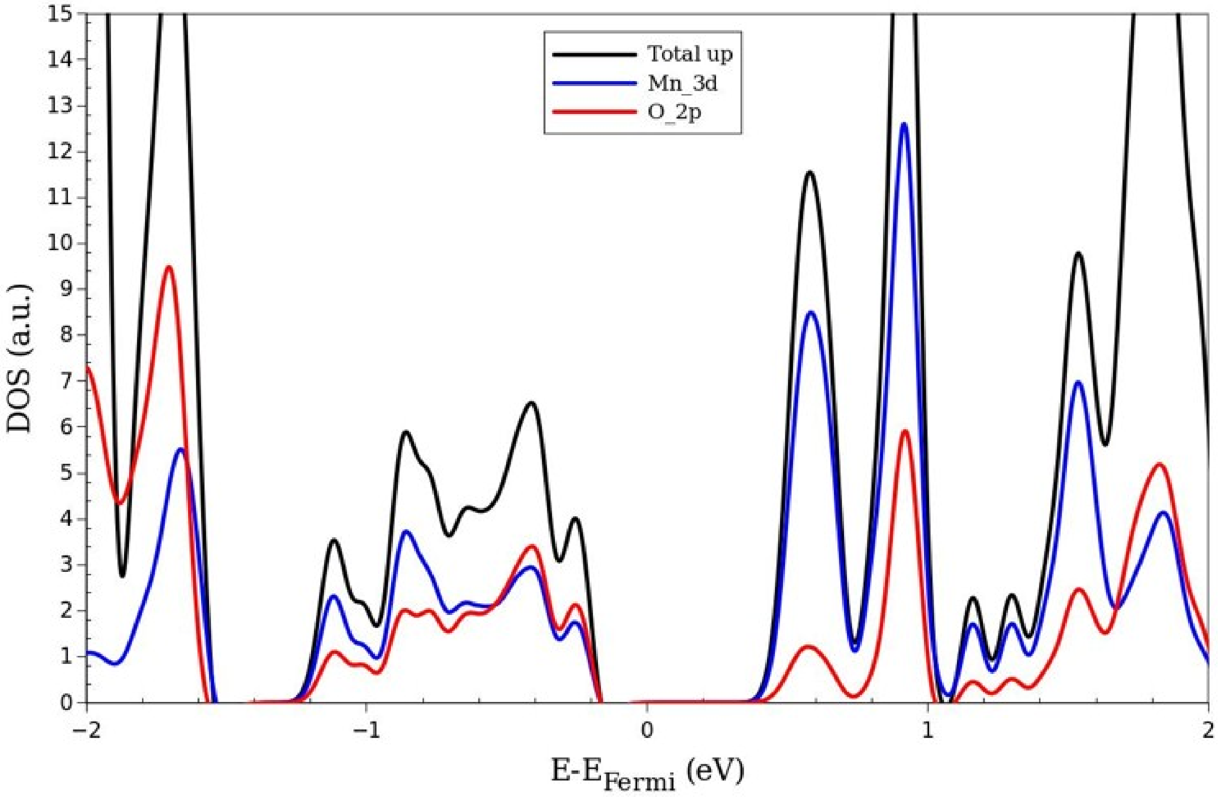

Figure S2. Total and projected DOS of large pore AF_b magnetic configuration

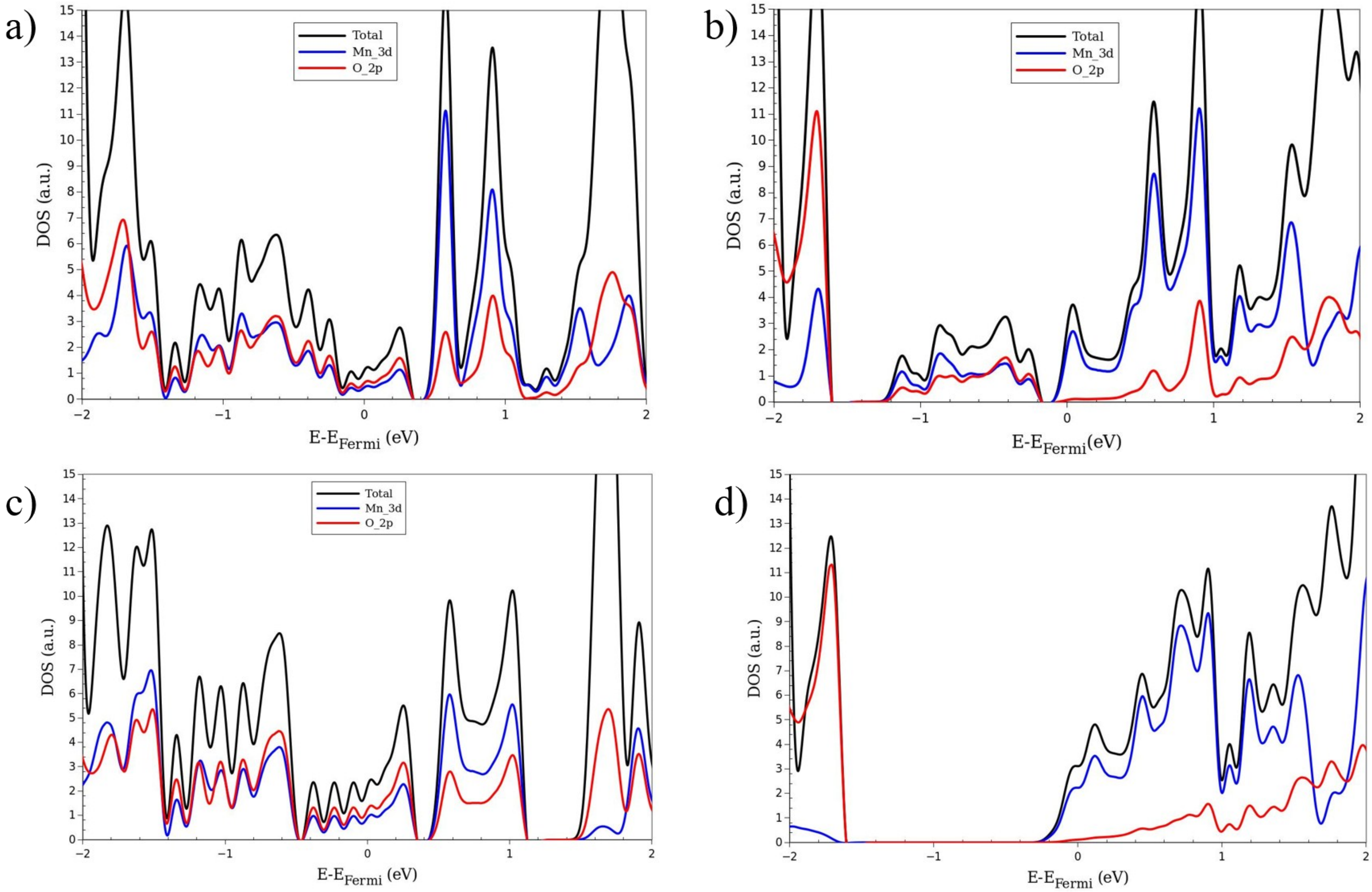

Figure S3. Total and projected density of states of large pore geometry in a) MIX spin up b) MIX spin down c) FM_a spin up and d) FM_a spin down magnetic configurations

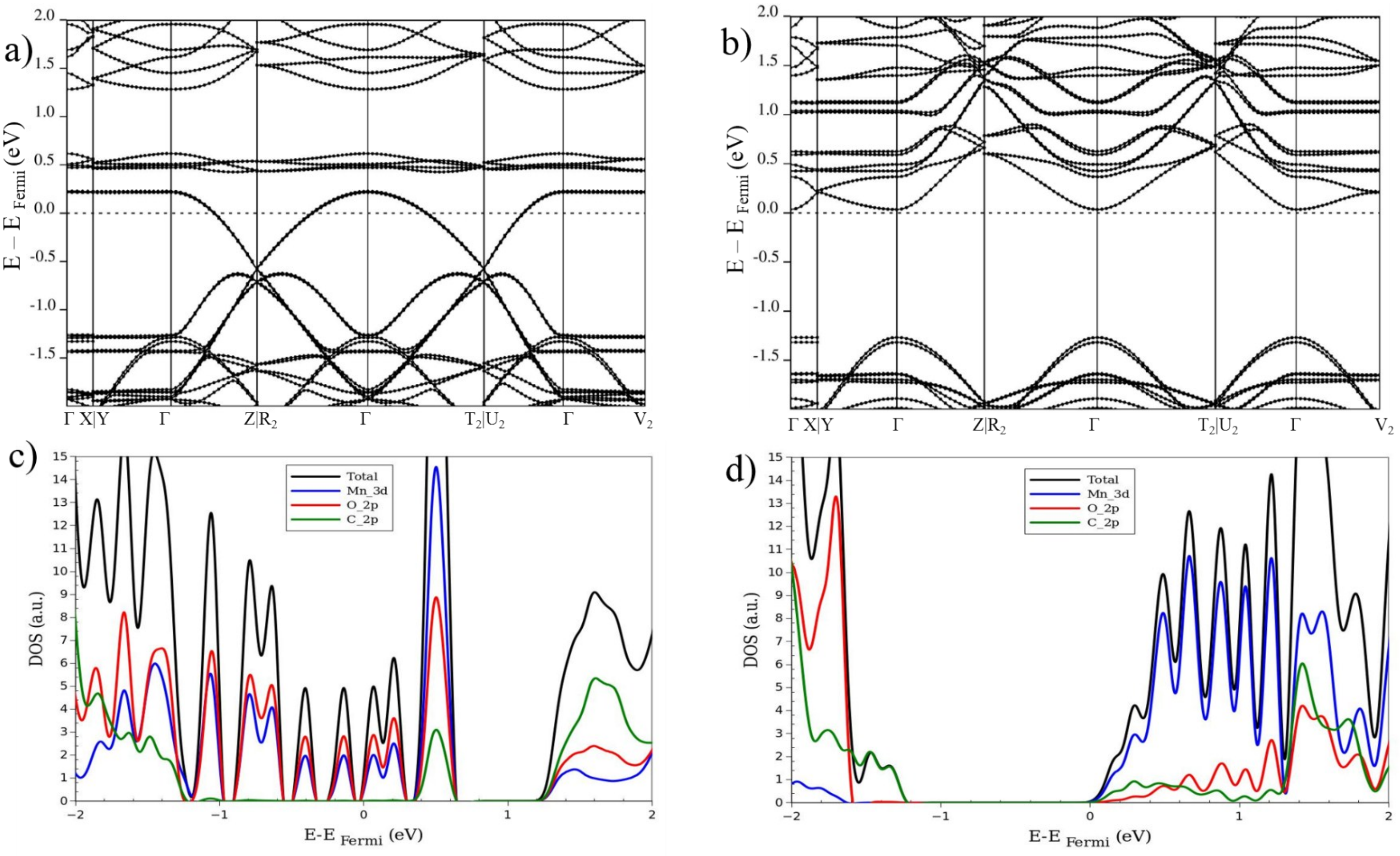

Figure S4. Electronic band structure of a) spin up and b) spin down for $FM_{a.}$ and their corresponding DOS and PDOS, c) spin up and d) spin down

S3. Charge density

Table S2. Hirshfeld-I charges of MIL-47 (Mn)

Atoms	Narrow Pore				
	FM_a	FM_b	AF_a	AF_b	MIX
Mn	2.394	2.391	2.392	2.391	2.393
O_c	-0.733	-0.732	-0.730	-0.731	-0.731
O_{Mn}	-1.155	-1.143	-1.157	-1.156	-1.158
C_H	-0.081	-0.081	-0.081	-0.080	-0.080
C_c	-0.108	-0.110	-0.109	-0.109	-0.109
C_o	0.855	0.851	0.854	0.854	0.854
H	0.131	0.130	0.130	0.130	0.130
	Large Pore				
Mn	2.404	2.393	2.400	2.400	2.402
O_c	-0.734	-0.735	-0.731	-0.731	-0.733
O_{Mn}	-1.160	-1.125	-1.158	-1.158	-1.158
C_H	-0.079	-0.080	-0.079	-0.079	-0.079
C_c	-0.101	-0.101	-0.102	-0.102	-0.102
C_o	0.844	0.837	0.842	0.842	0.843
H	0.130	0.129	0.129	0.129	0.129

S4. Atomic positions of structures

This section presents the atomic positions of both LP and NP materials (XYZ-format) corresponding to the lattice systems which are listed in table 1. Coordinates are in angstrom.

Large pore

Mn	12.131455286	10.406000547	1.626757012
Mn	12.127507649	10.406737641	4.873943310
Mn	4.031075074	3.468934732	1.624430383
Mn	4.027122188	3.468201366	4.871604617
O	10.731405744	9.140220545	2.100626405
O	2.627095177	2.202386103	5.345459193
O	13.527431922	9.140426624	4.400324623
O	5.431049038	2.202615263	1.150774669
O	2.652047325	4.756684732	4.387030932
O	10.756412454	11.694512864	1.142165308
O	5.405240798	4.758316549	2.109256073
O	13.501669344	11.696074727	5.358741225
O	5.427078449	4.734535814	4.397967346
O	13.531428458	11.672308133	1.153110537
O	2.631008195	4.734742774	2.098295863
O	10.727496840	11.672560591	5.347774984
O	13.505596665	9.116637951	2.111582166
O	5.401285530	2.178851112	5.356420340
O	10.752453055	9.118283634	4.389384754
O	2.656014425	2.180431344	1.139833290
O	12.125667827	9.783102236	6.497382422
O	4.029250536	2.845281884	3.247885099
O	4.025288575	4.091875616	6.495051357
O	12.129636494	11.029677813	3.250220187
C	8.553379982	7.450951301	2.036777917
C	0.449118366	0.513119513	5.281600178
C	15.702017817	7.446502394	4.464913226
C	7.605654489	0.508657859	1.215391869
C	0.571857315	6.562018761	4.458520805
C	8.676131787	13.499859644	1.213676647
C	7.482775452	6.566688170	2.038477449
C	15.579247222	13.504423220	5.287999531
C	7.601687360	6.428501275	4.462562872
C	15.706027896	13.366257700	1.217727454
C	0.453072511	6.423967275	2.034414902
C	8.549504944	13.361909232	5.283935774
C	15.583117983	7.308311317	2.040829268
C	7.478912781	0.370565632	5.285656158
C	8.672173936	7.312903825	4.460864863
C	0.575786395	0.375096175	1.211345086
C	9.153363673	7.827814000	3.247702673
C	10.303821140	8.776216823	3.245848980
C	1.056953487	0.889960367	-0.001821737
C	2.199559021	1.838418126	6.490728068
C	15.102591344	7.823918482	3.253885890
C	13.953835674	8.774504340	3.255506669
C	7.006191382	0.886163286	0.004361121
C	5.857391000	1.836757159	0.006029113
C	1.052948921	6.047185900	3.245371383
C	2.203474199	5.098710425	3.243515044
C	9.157363701	12.984998312	0.000528303
C	10.299921507	12.036565626	6.493064996
C	7.002212756	6.051040797	3.251558040
C	5.853428627	5.100418004	3.253175220
C	15.106565031	12.988789455	0.006715492
C	13.957800632	12.038199199	0.008363247
H	8.940447760	7.865717560	1.102886484
H	0.836189432	0.927864023	4.347717105
H	15.315055458	7.861240967	5.398801869
H	7.218600607	0.923405905	2.149297946
H	1.055762051	6.259608156	5.390474491
H	9.160093851	13.197346705	2.145654334
H	6.998626060	6.264538082	1.106535364
H	15.095107883	13.202265390	4.356047065
H	7.214688707	6.013718550	5.396476588
H	15.319012464	12.951486385	2.151640715
H	0.840077139	6.009253318	1.100555982
H	8.936615213	12.947170950	4.350046936
H	15.099016908	7.610414655	1.108883132
H	6.994769346	0.672757532	4.353696863
H	9.156157099	7.615370872	5.392822024
H	1.059740557	0.677558403	2.143299045

Narrow pore

Mn	20.986348277	-0.000789618	-0.015602314
Mn	19.699988212	6.506535187	2.938809214
Mn	7.916752930	3.249609615	5.912898360
Mn	9.203188618	3.256291151	2.958419053
O	8.313060463	1.847472701	1.939071839
O	10.088454364	4.663909745	3.980929107
O	11.374758661	1.841645691	1.026489956
O	7.026436471	4.658000015	4.893257771
O	18.814702642	5.098814447	1.916266743
O	-0.393606277	1.401003071	3.966209594
O	0.892447210	5.104519053	1.011614668
O	17.528348121	1.407089439	4.870705172
O	6.254715601	2.243199178	5.668820477
O	12.155820540	4.249680822	0.249567232
O	10.869437760	2.255817105	3.204074518
O	7.540958934	4.262263392	2.714112670
O	16.747220399	5.513079817	5.647782567
O	1.664653000	1.005208542	0.236351912
O	0.378143691	5.500138340	3.190684132
O	18.033632327	0.992907950	2.693245745
O	19.056926766	5.835527222	4.417678180
O	20.343405227	0.670332612	1.463190664
O	8.559797682	2.578448041	4.434130512
O	9.846166550	3.927299932	1.479544211
C	6.605169514	1.681175053	0.317300387
C	11.803449023	4.818079686	5.595977130
C	13.089861535	1.687298408	2.641678259
C	5.318456487	4.823357210	3.271393457
C	17.099694793	4.944215730	0.301251865
C	1.314412542	1.566504653	5.587938459
C	2.600442839	4.938340735	2.633437224
C	15.813106360	1.561007391	3.255556220
C	5.656374422	1.441071631	1.323632522
C	12.735864490	5.134503376	4.595481974
C	14.021825735	1.370698672	1.640904834
C	4.369606975	5.063516556	4.277940921
C	16.167393513	4.627760335	1.301747051
C	2.263216631	1.806481895	4.581649394
C	3.549193066	4.698055444	1.626870277
C	14.881239224	1.877547653	4.256389430
C	4.305654859	1.386007221	1.006778443
C	14.089057089	5.195915163	4.899938433
C	15.375638392	1.309376949	1.945630796
C	3.018957221	5.118519850	3.960953812
C	14.814254177	4.566340700	0.997286377
C	3.613808444	1.861521377	4.898486803
C	4.899847450	4.643047640	1.943875412
C	13.527366313	1.938866207	3.951659477
C	8.026958901	1.915807119	0.696246239
C	10.380175169	4.584366313	5.222255558
C	11.666461226	1.921217221	2.267882449
C	6.740263611	4.589251429	3.650428579
C	18.523166076	5.178283172	0.675020932
C	-0.107534703	1.332349345	5.209013341
C	1.178506744	5.173006953	2.254440012
C	17.236714287	1.327324401	3.629328558
H	5.998706524	1.315516007	2.354223412
H	12.379487069	5.313279351	3.577487618
H	13.665744748	1.191838788	0.623186193
H	4.712048867	5.189180950	5.308391510
H	16.523775529	4.449018156	2.319772644
H	1.920823743	1.932039457	3.550994348
H	3.206775028	4.572435255	0.596412381
H	15.237282067	2.056420001	5.274078581
H	3.559734962	1.189363466	1.779970682
H	14.820736379	5.454129209	4.130972341
H	16.106944172	1.051093217	1.176542585
H	2.273041004	5.315319447	4.734294410
H	14.082518472	4.308132947	1.766265224
H	4.359815638	2.058176239	4.125268620
H	5.645784771	4.446277675	1.170539535
H	12.796092338	2.197124330	4.720717670

S5. Projected DOS of Mn atom

Here, we present the Projected DOS for Mn atoms with different m_l to support the +4 oxidation state.

a)

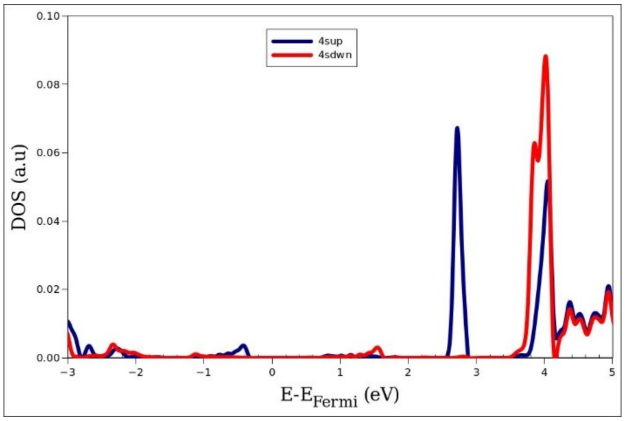

b)

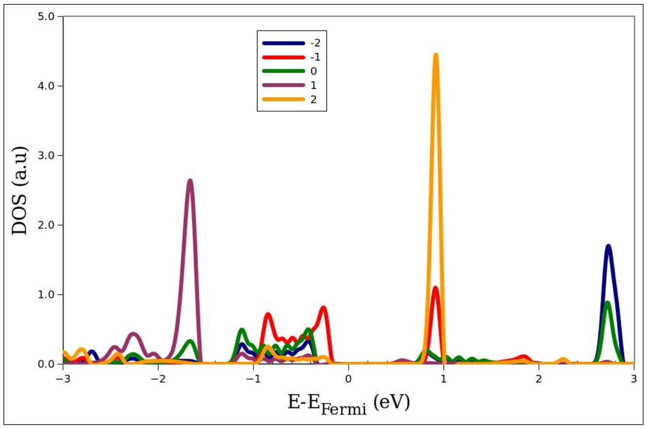

c)

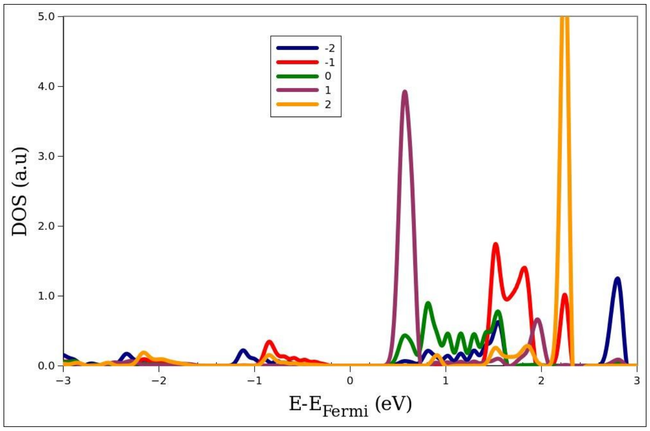

Figure S5. Projected DOS for one of Mn atoms in AF_a magnetic configuration for large pore geometry. a) for 4s electrons b) 3d electrons spin up c) 3d electrons spin down. Note that the m_l numbers follow this order: -2, -1, 0, +1 and +2 correspond to $d_{x^2-y^2}$, d_{z^2}, d_{xy},d_{xz}, d_{yz}.

As one can see, the sharp peak for 4s electrons are far from Fermi level showing ionization of these electrons. For 3d electrons, presence of three peaks below the Fermi level and two peaks above this for spin up and lack of any significant DOS below the Fermi level for spin down, confirms the +4 oxidation state in this material.
